# Metabolism of inositol derivatives by the gut microbiome

**DOI:** 10.1080/19490976.2025.2564765

**Published:** 2025-09-29

**Authors:** Henning Jessen, Thi Phuong Nam Bui

**Affiliations:** a Institute of Organic Chemistry & Centre for Integrative Biological Signaling Studies (CIBSS), University of Freiburg, Freiburg, Germany; b Department of Experimental Vascular Medicine, Amsterdam University Medical Center, Amsterdam, the Netherlands

**Keywords:** The gut microbiome, inositol, inositol phosphates, phytate, short chain fatty acids, phytase

## Abstract

The gut microbiome harbors immense metabolic capacity, much of which is still being uncovered. Recent discoveries have highlighted the ability of gut microbes to metabolize inositol-derived compounds, including free inositols and their phosphorylated derivatives, inositol phosphates. While these compounds are abundant in plant-based diets, our understanding of the specific bacterial taxa involved and their metabolic pathways has only recently begun to emerge. Intriguingly, inositols and inositol phosphates have been associated with a range of health benefits. Although some effects have been attributed to direct absorption or chemical interactions within the host, increasing evidence points to the gut microbiota as a key mediator in unlocking their therapeutic potential. This review emphasizes the microbial metabolism of inositol derivatives, highlights the intersection between diet, microbial function, and host health, and discusses their implications for nutrition and future therapeutic strategies.

## Introduction

The human gastrointestinal (GI) tract harbors trillions of microbial cells, forming a dynamic ecosystem that functions as an integral organ. These microbes play critical roles in maintaining health and modulating disease processes.^1-3^ Alterations in the composition and function of the gut microbiome, often described as gut microbiota disruption, are frequently associated with various health issues.^4^^,^^5^ A central function of the gut microbiome is the metabolism of dietary and host-derived components, leading to the production of bioactive compounds such as short-chain fatty acids (SCFAs), vitamins, indoles, and secondary bile acids, all of which are essential regulators and mediators of host physiology.^6-10^ SCFAs have gained significant attention as they are signaling molecules that have been shown to mediate histone deacetylation and cell proliferation^11^ and activate G protein-coupled receptors such as GPR41 and GPR43,^12^^,^^13^ contributing to the management of obesity and diabetes^7^ and suppressing colonic inflammation and carcinogenesis.^14^

Diet is a major determinant of gut microbiome composition and function, thereby significantly influencing human health.^15-17^ Diets rich in whole, minimally processed foods support a diverse and balanced microbiome, whereas excessive consumption of processed foods and refined sugars is associated with reduced microbial diversity and increased health risks of chronic diseases.^18^^,^^19^ In 2018, suboptimal diets were estimated to contribute to approximately 70% of new type 2 diabetes cases worldwide.^20^ Notably, inadequate whole grain consumption was linked to a 26% increase in type 2 diabetes and cardiovascular disease incidence, which remain among the most prevalent metabolic disorders in modern populations. A recent longitudinal study of more than 100,000 participants followed for up to 30 y demonstrated that higher intakes of fruits, vegetables, whole grains, nuts and legumes were correlated with healthier ageing outcomes.^21^ These foods not only provide indigestible polysaccharides such as fiber, resistant starch, and inulin but also contain bioactive compounds such as phenolics, antioxidants, and inositol phosphates, particularly phytate (InsP_6_).

Considerable research focused on the gut microbial metabolism of fibers^22-24^ and polyphenols^25^ demonstrate health benefits of fibers through the modulation of the gut microbiome. Multiple studies have consistently reported that high dietary fiber intake is associated with increased microbial diversity and a greater abundance of fiber-degrading bacteria, including *Prevotella,*^26^ *Bifidobacterium*,^27^ *Roseburia,*^28^ and *Ruminococcus*.^26^ These fiber-degrading bacteria can directly produce short-chain fatty acids^29^ or crossfeed with butyrogenic species such as *Eubacterium* and *Anaerostipes*, leading to increased production of beneficial SCFAs like butyrate and propionate.^22^^,^^30^ In addition, a fiber-rich diet increases the intestinal transit time,^31^ promoting the production of fermentation metabolites and reducing the absorption or interaction of potentially harmful compounds.^32^ More recent studies have shown that fibers influence microbial tryptophan metabolism, favoring the production of protective indole derivatives such as indolelactic acid (ILA) and indolepropionic acid (IPA) through microbial interactions within the gut microbiota.^33^ Interestingly, various bioactive food components in vegetables are also metabolized by the gut microbiome, contributing further to health benefits.

Inositols and their derivatives are abundant components of both the human diet and mammalian cells.^34^^,^^35^ Studies on intracellular inositol metabolism have highlighted the roles of inositol derivatives in cell signaling and regulation, protein structure, cancer metastasis and cell proliferation.^36-38^ Despite the regular consumption of these compounds, our understanding of how gut bacteria utilize inositol derivatives for growth, energy conservation and lipid synthesis and how these processes influence gut homeostasis and systemic health has only recently been uncovered. This review explores the gut microbes involved in the metabolism of inositol derivatives, the associated catabolic and anabolic pathways, and their potential implications for human health.

## Dietary inositol derivative intake and its absorption/secretion

Inositol derivatives (mostly *myo*-configuration) encompass a broad class of molecules, including free inositols, inositol phosphates and phosphatidylinositols (PIs), each with distinct biological roles and dietary sources. Free inositols and inositol phosphates (such as phytate) are found primarily in the cytosol of plant cells and are abundant in plant-based foods. In contrast, phosphatidylinositols are membrane-bound lipids that play essential roles in signal transduction and membrane structure, predominantly within eukaryotic cellular membranes. Notably, the synthesis of PIs has only recently been reported in gut bacteria.^39^

** Inositols** are naturally occurring carbohydrates abundantly present in the human diet, particularly in plant-derived foods, where they are predominantly found in the form of inositol phosphates. Nine stereoisomers of inositol exist in nature: *epi, cis-, neo-, allo-, scyllo-, muco-, myo-, D-chiro-* and *L-chiro-*Inositol, with *myo-*inositol being the most prevalent and biologically significant isomer.^40^ Among these, only a few exhibit distinct physiological roles, with *myo*-inositol playing a central role in cellular metabolism. Inositol uptake occurs through both sodium ion-coupled and proton-coupled transport mechanisms.^41^ However, the primary route for *myo*-inositol transport in mammalian cells, including enterocytes, is mediated via the sodium cotransporters SMIT1 and SMIT2, which facilitate active absorption in the small intestine.^42^^,^^43^ Interestingly, these transporters have also been implicated in glucose uptake into enterocytes, highlighting their dual substrate specificity. Pharmacokinetic studies in rats have shown that *myo*-inositol appears in the serum within a few hours of oral administration, followed by a gradual decline over 24 h, maintaining levels slightly above baseline.^44^ In addition to host absorption, inositols may also serve as substrates for microbial fermentation in the gut, as explored later in this review.

** Inositol phosphates** represent the primary natural source of phosphorus in plant-based diets, encompassing a range of compounds with phytate (*myo*-inositol hexakisphosphate, InsP₆) as the most abundant form, particularly prevalent in seeds and grains.^45^ The phytate content varies between 0.14% and 2.05%, contributing to approximately 18%–88% of the total phosphorus content in cereals and legumes.^46^ Dietary phytate intake differs considerably across countries and demographic groups, including sex and age.^34^ In infants aged 6−12 months, the estimated daily intake ranges from 26 to 189 mg,^47^ increasing with age up to 3380 mg in children aged 7−9 y.^48^ In adulthood, phytate intake is largely shaped by dietary habits and food culture. In developing countries, where cereal and legume consumption is high, daily phytate intake may reach up to 2 g, whereas in industrialized nations, it typically remains below 600 mg.^34^ Vegetarian populations, particularly lacto-ovo vegetarians, often exhibit substantially higher phytate intake, with reported values reaching up to 6 g due to the predominance of plant-derived foods in their diets.^49^

A pharmacokinetic study using radiolabelled C^14^-phytate in mice revealed that oral administration of phytate did not result in the production of any inositol phosphate derivatives other than free inositol within the first 24 h.^50^ In contrast, intravenous injection of phytate led to the rapid accumulation of various inositol phosphate intermediates, including InsP_6_, InsP_5_, InsP_4_, InsP_3_, InsP_2_, InsP, and free inositol, within 5 min, which were subsequently metabolized into inositol. This dephosphorylation of InsP_6_ to lower inositol phosphates may be catalyzed by phosphatases present in plasma.^51^ Notably, hepatic inositol levels in orally treated mice were approximately tenfold lower than those in intravenously injected mice. These results suggest that orally administered phytate was primarily metabolized in the gut, likely by the microbiota, and is absorbed in the form of inositol rather than intact phytate. Similarly, a study in pigs demonstrated that more than 97% of dietary phytate was utilized in the gastrointestinal tract, regardless of the animal’s intrinsic phytase levels,^52^ reinforcing the role of gut bacteria in phytate degradation. This finding aligns with evidence from human studies showing poor systemic absorption of phytate, showing similar urinary phytate excretion in healthy volunteers regardless of whether they ingested 400 mg, 1400 mg or 3200 mg of phytate.^53^ Furthermore, in a Korean cohort, more than 70% of dietary phytate appeared to be metabolized in the gut,^54^ with fecal phytate excretion correlated with dietary intake in younger individuals but not in elderly individuals. Another study suggested that urinary phytate excretion correlated with phytate intake in individuals consuming Mediterranean dietsx;^55^ however, the quantification of phytate based on phosphorus levels may overestimate phytate due to the presence of multiple inositol phosphate derivatives that can coelute during chromatographic purification. This highlights the need for more accurate analytical methods, such as capillary electrophoresis‒mass spectrometry,^56^ especially considering the inability of phytate to cross the intestinal epithelium.^34^

Despite the variation in phytate excretion reported across human studies, there is a general consensus that dietary phytate is primarily metabolized in the gut. Population-level differences in phytate excretion likely reflect both cultural dietary pattern and interindividual variation in the gut microbiome composition and metabolic capacity. Diet is a well-established driver of microbiome composition and functions,^57^^,^^58^ and this assumption is further supported by previous observations showing that the abundance of phytate-degrading bacteria varies by ethnicity.^59^

## Inositol fermentation by gut bacteria

**Figure 1.**
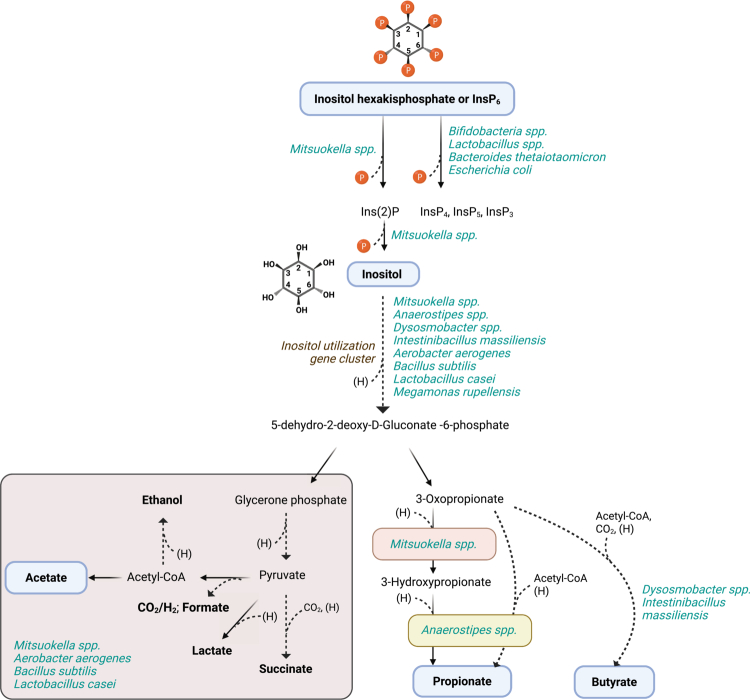
Postulation of microbial inositol and phytate degradation pathway in the human gut. Dashed row: multiple steps. The gray box for the reaction steps found in particular bacterial species at the bottom left. Figure created with BioRender.com.

To date, several bacterial species have been reported to ferment *myo*-inositol and phytate to various end products (Figure 1). Inositol fermentation was first characterized in the gut isolate *Aerobacter aerogenes,* which converts *myo*-inositol via a *keto*-inositol route to ethanol, acetate, succinate, CO_2_ and H_2_ under anaerobic conditions.^60^ Subsequent studies reported the aerobic conversion of *myo*-inositol to dihydroxyacetone phosphate, acetyl-CoA and CO_2_ in the soil bacteria *Bacillus subtilis*^61^ and *Lactobacillus casei* BL23.^62^ It has been shown that soil bacteria predominantly take up *myo*-inositol mostly via the transporter iolT,^63^ which has also been identified in other commensals.^64^ The catabolism of *myo*-inositol is initiated by its conversion through an inositol cascade to dihydroxyacetone, a process biochemically and genetically characterized in both *L. casei* (45) and *B. subtilis*. This pathway involves the iolABCDEFGHIJ operon for inositol utilization (44). However, it is important to note that these bacteria typically degrade *myo*-inositol under aerobic conditions and are not native members of the human gut microbiome.

Recently, it was discovered that *Anaerostipes* spp., an abundant butyrogenic commensal in the human gut, are capable of converting *myo*-inositol to the short chain fatty acids (SCFAs) propionate and acetate.^64^ Proteogenomic analysis of *Anaerostipes rhamonosivorans* revealed the presence and functional expression of *myo*-inositol dehydrogenase and epi-inositol hydrolase, enzymes involved in the conversion of a less abundant inositol isomer, D-*chiro*-inositol to scyllo-isomerase. Interestingly, *Anaerostipes* spp. utilized D-c*hiro*-inositol at a significantly slower rate compared to *myo*-inositol, likely reflecting limited dietary exposure to minor inositol isomers. Pathway analysis revealed the presence of an inositol gene cluster and a newly identified CoA transferase, which plays a critical role in the conversion of *myo*-inositol to propionate. Phylogenetic analysis of CoA transferases indicated that *Anaerostipes* employs a 3-oxoacid CoA transferase gene for propionate formation and a butyryl-CoA:acetate CoA transferase for butyrate formation, underlying its versatile metabolic features to produce a range of beneficial metabolites. It has been shown that inositol fermentation contributes to energy conservation through both substrate-level phosphorylation and electron transport phosphorylation.

Notably, not all *Anaerostipes* species possess the genetic capacity to metabolize *myo*-inositol, and this difference is also observed at the strain level.^64^ Among the tested *Anaerostipes hadrus* strains, three strains lacked both the ability to utilize *myo*-inositol and the genes encoding the inositol metabolic pathway, whereas two strains harbored the full pathway and successfully converted *myo*-inositol into SCFAs. These findings underscore the strain-level variability and metabolic adaptability of gut bacteria in response to specific diets. In the same study, genomic analysis of over 10,000 NCBI genomes revealed that seven bacterial genomes contained the complete inositol pathway, five of which belong to the *Anaerostipes* genus, highlighting their central role in *myo*-inositol-to-propionate conversion in the gut. The discovery of this pathway adds a fourth propionate biosynthesis route to known synthesis pathways, including acrylate, methylmalonyl-CoA, and propanediol pathways.

Emerging evidence suggests that certain gut bacteria are capable of converting *myo*-inositol to butyrate,^65^ although the precise metabolic pathway underlying this transformation has yet to be fully characterized. Notably, species such as *Dysosmobacter welbionis* and *Intestinibacillus massilensis* (unpublished data) appear to be involved in this conversion. Further studies are needed to elucidate the enzymatic steps and regulatory mechanisms of this *myo*-inositol-to-butyrate fermentation pathway.

It was recently reported that *Mitsuokella* spp. is capable of converting *myo*-inositol to 3-hydroxypropionate, an antimicrobial compound and a key metabolic intermediate in the gut.^59^ The production of this compound may enhance the competitive fitness of *Mitsuokella* by providing a selective advantage in the densely populated gut environment. Indeed, culture supernatants of *Mitsuokella jalaludinii* have been shown to inhibit the growth of *Salmonella enterica* and suppress the expression of its virulence factors.^66^

## Dietary inositol phosphate metabolism by gut bacteria

### Initial dephosphorylation of phytate by microbial phytases

Unlike inositols, dietary inositol phosphates serve as reservoirs for both inorganic phosphate and fermentable carbon for microbial fermentation in the gut. The microbial metabolism of dietary inositol phosphates, particularly phytate, represents a critical yet understudied aspect of diet‒microbiota‒host interactions. To utilize phytate, bacteria must first hydrolyse it into lower phosphorylated inositol phosphates and free phosphate via the action of phytases. These enzymes are classified based on their initial site of cleavage on the inositol ring: 3-phytases (C1 or C3), 6-phytases (C6), and 5-phytases (C5). While 3-phytases are commonly found in bacteria and fungi,^67^^,^^68^ 6-phytases are generally of plant origins (e.g., grains, seeds), and 5-phytases have been isolated from legumes and ruminal bacteria *Selenomonas ruminantium*.^69^

Several anaerobic ruminal bacteria have been reported to exhibit strong phytase activity, including *Selenomonas ruminatium* and *Mitsuokella* species.^70^ *S. ruminantium* hydrolyses phytate through a sequential reaction using a 5-phytase, which shows no sequence homology with known microbial phytases.^71^ Other ruminant bacteria, such as *M. jalaludinii* and *Mitsuokella multiacidus,* have also demonstrated high levels of phytase activity.^72^^,^^73^ However, the specific degradation products resulting from their phytate hydrolysis remain unidentified. In addition, the soil bacterium *B. subtilis* has also been noted for its robust phytase activity.^74^ In contrast, fungal and mammalian phytases typically hydrolyze phytate to produce lower phosphorylated *myo*-inositol derivatives, such as a human phytase (MINPP1) and a 3-phytase (phyA) produced *by Aspergillus niger* to generate such intermediates.^75^

**Figure 2.**
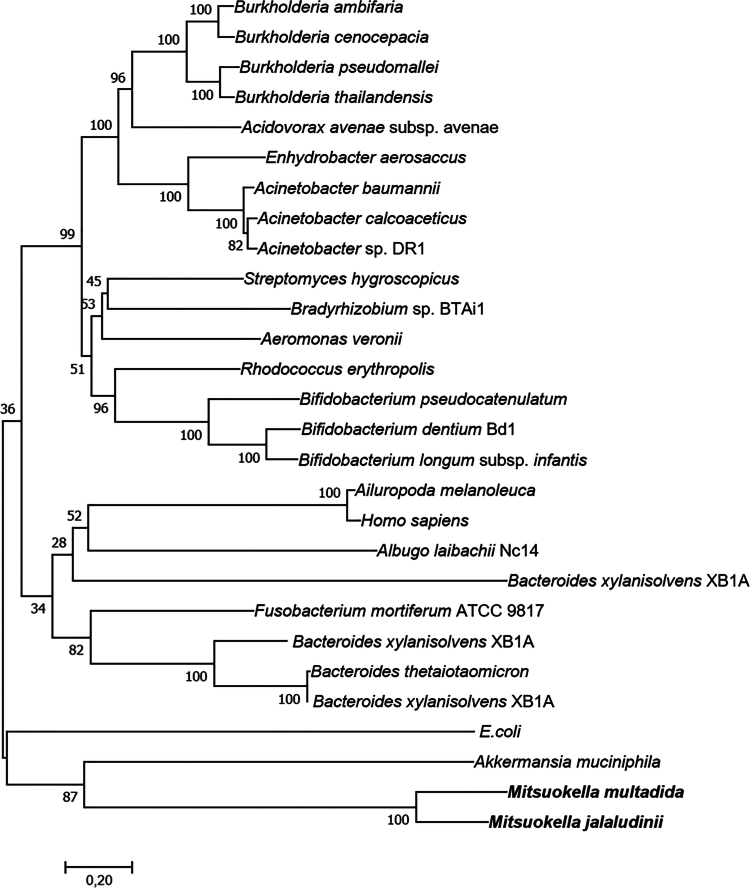
Phylogeny of phytase. A phylogenetic tree constructed using predicted phytases from *M. jalaludinii* and *M. multacida* (denoted in bold), along with representative bacterial and mammalian phytases using the maximum likelihood algorithm. Amino acid sequences were retrieved from the NCBI database using accession numbers reported in a previous study.^82^ The scale bar indicates 20**%** sequence divergence.

Although numerous gut bacteria have been reported to exhibit phytase activities, their capacities to hydrolyse phytate vary significantly. For example, *Bacillus spp.*, *Escherichia coli*, *Bifidobacterium* spp., and *Lactobacillus* spp. degrade phytate only partially,^76-79^ producing a range of inositol phosphate derivatives while *Mitsuokella* spp. are capable of complete dephosphorylation of phytate to *myo*-inositol.^59^^,^^72^^,^^73^ Some bacteria, particularly *Bifidobacteria* and *Lactobacillus* species, have been shown to convert approximately 6% of total InsP₆ into a mixture of InsP_3_, InsP₄, and InsP₅.^77^^,^^80^ Notably, bacterial phytases function across a broad pH range (2.5–7.5),^81^ indicating their potential to degrade phytate in the upper gastrointestinal tract. The extent of dephosphorylation and pH optima can vary among *Bifidobacterium* species.^76^ *Bacteroides thetaiotaomicron* expresses a 3-phytase (btMINPP) that hydrolyzes phytate to Ins(1,4,5)P_3_ and Ins(1,3,4)P_3_,^82^ while *E. coli* phytase, which functions as a 6-phytase, generates multiple InsP_3_ isomers, such as Ins(2,4,5)P_3_, Ins(1,2,3)P_3,_ Ins(1,2,4)P_3_ and Ins(1,2,5)P_3_.^78^ Similarly, phytases from other *Bacillus* spp. have been reported to produce alternative isomers of InsP_3_, including Ins(2,4,6)P_3_ and Ins(1,3,5)P_3,_^79^ and the structures of these enzymes have been elucidated.^83^ To date, *M. jalaludinii* remains the only human gut isolate capable of fully dephosphorylating InsP₆,^59^ while a the complete conversion of phytate to SCFAs by the fecal microbiome is a common feature.^59^^,^^64^ This warrants further investigation of unknown phytate degraders in the gut of individuals who do not harbor *Mitsuokella* in their microbiomes. This complete dephosphorylation proceeds via two steps: the initial hydrolysis of InsP₆ to Ins(2)P, followed by further dephosphorylation to release free inositol, which can subsequently be utilized by bacteria for fermentation. Phylogenetic analysis indicates that *Mitsuokella* phytases are distantly related to those from other gut bacteria or mammalian systems, potentially explaining their unusually high catalytic efficiency (Figure 2). Furthermore, the cloning of a phytase gene from the ruminant-derived *M. jalaludinii* strain into *E. coli* revealed that the enzyme exhibits activity across a pH range of 3.5–5.5, with an optimal pH of 4.5.^73^ The enzyme activity was enhanced by Ca^2+^, Mg^2+^, and K^+^, while inhibited by Cu^2+^, Fe^3+^, and Zn^2+^. Whether phytases from human isolates possess similar biochemical characteristics remains to be elucidated.

Due to its highly polyanionic nature, phytate is unable to cross bacterial membranes. Consequently, bacteria must either secrete extracellular phytase or anchor the enzyme in their outer membrane to access phytate. Certain gut bacteria, such as *B. thetaiotaomicron*, have been shown to secrete phytase encapsulated in extracellular vesicles, leading to phytase activity in the culture supernatant while no activity was detected in the cellular pellet.^82^ These versicles have been proposed to function as delivery systems, transporting dephosphorylation products like InsP_3_ to host enterocytes. Similarly, *E. coli* also exhibited phytase activity in the culture supernatant, suggesting enzyme release into the environment.^78^ In contrast, electron microscopy studies have shown that species like *Mitsuokella multidacidus* and *S. ruminatium* possess membrane-anchored phytases.^72^ Consistently, high phytase activity in cell lysates but absence in the supernatant of *S. ruminatium* supports this membrane-associated localization.^69^ Supporting this, transmembrane helix prediction using the TMHMM model indicates that the phytase produced by *M. jalaludinii* is likely membrane-integrated. Beyond low sequence homology with other known phytases, *Mitsuokella* spp. are among the few gut species with membrane-bound phytases, which may underpin their strong phytate-degrading capacity. These findings suggest that phytase localization plays a critical role in the efficiency of phytate dephosphorylation. The complete hydrolysis of phytate by gut bacteria could enhance inositol bioavailability for both the host and microbes while also minimizing nutrient loss associated with incomplete phytate degradation. This process could potentially improve the nutritional value of plant-based diets. To date, *Mitsuokella* spp. are the only characterized gut bacteria capable of complete phytate dephosphorylation and subsequent fermentation of released inositol,^59^ leaving little to no inositol available for epithelial absorption and membrane metabolism. However, because inositols are primarily absorbed in the upper gastrointestinal tract,^42^ while microbial phytate degradation is more likely to occur in the large intestine,^59^ the impact of complete bacterial phytate degradation on host inositol availability may be limited. Future studies are needed to clarify this issue and to determine whether additional intestinal phytate degraders that do not utilize inositol also contribute to phytate metabolism in the human gut.

### SCFA production from microbial cross-feeding

Complete degradation of phytate by the human gut microbiota results in the production of SCFAs, primarily propionate, butyrate and acetate. Interestingly, distinct gut microbiomes exhibit divergent fermentative capacities, converting phytate to either propionate and acetate or butyrate and acetate.^59^^,^^64^ 16S rRNA gene–based community analysis revealed that these two metabolic routes are mediated by different bacterial taxa. For instance, analyses of human fecal phytate incubations showed that *Ruminococcaceae, Mitsuokella* and *Butyricicoccus* were enriched in samples exhibiting rapid phytate degradation with propionate and acetate production. In contrast, increased abundances of *Butyricicoccus, Mitsuokella* and *E. coli/Shigella* were observed under conditions of slower phytate degradation, associated with butyrate and acetate formation.^59^ These findings led to the identification of *Mitsuokella* as a prevalent phytate-degrading bacterium in the human gut. In the multiethnic prospective Helius cohort (*n* = 6039), *Mitsuokella* was present in approximately 30% of individuals with an abundance of up to 10%, although it is typically around 1% in most microbiomes. Notably, *Mitsuokella* was strongly associated with *Prevotella*, a well-known fibre-degrading genus commonly enriched in individuals consuming high-fiber diets.^84^ This co-occurrence likely reflects a coordinated microbiome response to two major dietary components—fibre and phytate—that naturally coexist in plant-based foods.

The microbial conversion of phytate to short-chain fatty acids within the gut microbiota requires metabolic cross-feeding between different bacterial species.^59,64^ Notably, known phytate-degrading bacteria like *Mitsuokella* spp. do not produce SCFAs directly from phytate, as evidenced by *the in vitro* conversion of phytate to lactate, succinate, 3-hydroxypropionate and acetate.^59^ Therefore, SCFA production from phytate likely necessitates metabolic interactions with other commensals. A striking example is the cooperative interaction between *M. jalaludinii* and *Anaerostipes rhamnosivorans*, which enables propionate production from phytate. Remarkably, this cross-feeding does not occur via interspecies transfer of inositol but rather via 3-hydroxypropionate – an antimicrobial compound. In monoculture, *M. jalaludinii* dephosphorylates phytate to release inositol, which is then fermented to 3-hydroxypropionate, succinate and acetate. Although *A. rhamnosivorans* grows more efficiently on inositol in monocultures, the presence of 3-hydroxypropionate inhibits its inositol fermentation, allowing *M. jalaludinii* to dominate inositol utilization. Subsequently, *A. rhamnosivorans* uses 3-hydroxypropionate produced by *M. jalaludinii* to synthesize propionate. This metabolic interplay may serve as a detoxification strategy by converting the antimicrobial 3-hydroxypropionate into a growth-supporting metabolite, illustrating a sophisticated survival mechanism within the gut ecosystem. While the mechanisms underlying butyrate production from phytate remain unclear, it is plausible that *Mitsuokella* species also cooperate with butyrogenic commensals in a similar manner. Further studies are required to elucidate the metabolic pathways and interspecies interactions involved in this process.

## Inositol lipid metabolism

Gut bacteria not only use inositol phosphates as sources of carbon and energy but also incorporate them into the biosynthesis of membrane lipids, contributing to host‒microbe symbiosis.^39^ While the synthesis of inositol-derived lipids has been extensively characterized in yeast and mammalian cells,^85^ it has only recently been observed in members of the gut microbiome. Notably, *Bacteroides* spp. have been shown to synthesize sphingolipids, including ceramide phosphoinositol and deoxy-sphingolipids, which are thought to play a role in maintaining microbe‒host symbiotic relationship.^86^ Recent studies have demonstrated that *B. thetaiotaomicron* can synthesize *myo*-inositol phosphate via the *de novo* sphingolipid synthesis pathway. Loss of inositol lipids has been linked to alterations in bacterial capsule expression and antimicrobial resistance *in vitro* and decreased bacterial fitness in mice.^39^ Furthermore, a variant form of inositol lipid synthesis has been found to be widespread among *Prevotella* species, indicating a broader prevalence and functional significance of inositol-derived lipids across different gut microbial taxa.

## Health implications of microbial Inositol phosphate metabolism

### Health benefits from Inositol supplementation

Inositols have been reported to promote metabolic health and ovarian functions in patients with polycystic ovary syndrome (PCOS).^87^^,^^88^ These benefits are generally attributed to the systemic absorption of orally administered inositols, which act as insulin-sensitizing molecules within target cells, thereby enhancing cellular metabolism.^89^ However, the potential contribution of gut microbial activity to the observed effects of inositols in PCOS cannot be overlooked. Several gut bacteria are known to catabolize inositols to a variety of bioactive metabolites, including SCFAs such as propionate and butyrate, which have been shown to promote metabolic fitness.^7^ Notably, the administration of the inositol-utilizing *A. rhamnosivorans* improved glucose metabolism in mice, and this benefit was associated with enhanced fermentation of *myo*-inositol to propionate.^64^ Consistent with this, the presence of a structural genomic variant encoding inositol catabolic pathway genes from *A. hadrus* was associated with reduced metabolic risks in large human cohort studies.^90^ Conversely, a recent finding suggested that gut microbial *myo*-inositol degradation may also contribute to adverse metabolic outcomes under certain conditions. In this study, mice gavaged with the inositol-degrading strain *Megamonas rupellensis* exhibited increased lipid absorption and obesity, an effect attributed to the reduced availability of *myo*-inositol in the gut lumen.^91^ It remains to be clarified whether this outcome results primarily from decreased inositol uptake by enterocytes or from the metabolic effects of inositol-derived fermentation products. The latter possibility is plausible, as reduced SCFA production—particularly by beneficial commensals such as *Anaerostipes* spp.—may compromise the protective functions of the gut bacteria. Importantly, the health benefits of microbial inositol fermentation only complement those of epithelial absorption and metabolism, as inositols are primarily absorbed in the upper gastrointestinal tract,^42^ whereas microbial conversion predominantly occurs in the large intestine.^64^^,^^65^ Further investigation is required to elucidate the extent and physiological relevance of microbial inositol conversion relative to epithelial metabolism.

### Medicinal effects for dietary phytate – what do we know

**Figure 3.**
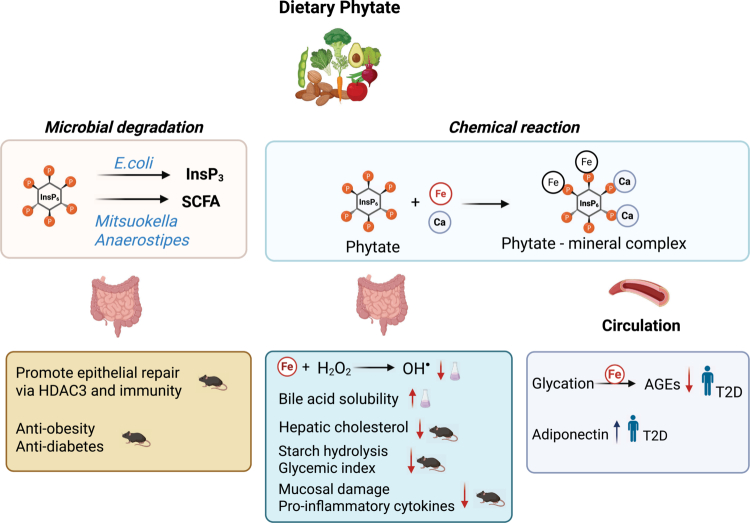
Overview of reported health effects of dietary phytate based on *in vitro* and *in vivo* models and human studies. Figure created with BioRender.com.

Phytate, a naturally abundant compound in plant-based diets, has been extensively studied for its potential health benefits (Figure 3). These benefits can be broadly attributed to two mechanisms: (i) direct chemical or physical interactions occurring locally in the gut or systemically and (ii) microbial metabolism.

The first mechanism stems from the polyanionic structure of phytate, which enables it to chelate metal ions such as iron. This chelation can inhibit iron-mediated hydroxyl radical formation *in vitro* at concentration of 100  µM and 500 µM,^92^ a property that has been proposed to contribute to the suppression of colonic carcinogenesis and inflammatory bowel diseases.^93^ However, such antioxidative effects have not been consistently demonstrated *in vivo*, as phytate supplementation did not significantly alter the antioxidant status in rats fed 1% phytate.^94^ Similarly, although phytate increases bile acid solubility *in vitro* when it is coincubated with up to 0.1% sodium phytate, *in vivo* supplementation of 0.4% phytate in the diet of rats led to increased bile acid secretion and elevated serum cholesterol levels without improving fat digestibility after 24 d.^95^ These discrepancies between *in vitro* and *in vivo* findings suggest the involvement of additional yet unidentified possibly microbial factors in modulating phytate’s physiological effects. Phytate has also been implicated in glycemic control. It is postulated to lower the glycemic index by inhibiting host amylases through calcium chelation.^96^ *In vitro*, the addition of 2 or 5% phytate reduced starch degradation by human saliva^97^ and ileostomy contents,^98^ respectively; this inhibitory effect was reversed upon calcium reintroduction.^96^ In human studies, supplementation of 380 mg calcium-magnesium phytate for 12 weeks increased adiponectin levels in individuals with type 2 diabetes,^99^ while animal studies reported improvements in intestinal barrier integrity and reductions in proinflammatory cytokines when rats were fed with either 0.25, 0.5 or 1 g/kg phytate daily for 38 weeks^100^ or 2.04% phytate or 1.02 phytate plus 0. 2 myo-inositol for 3 weeks.^101^

One hypothesis suggests that oral phytate may exert systemic effects via increased circulating phytate. For example, phytate supplementation was shown to reduce plasma advanced glycation end products (AGEs), potentially through ion chelation, a critical co-factor of AGE formation.^102^ However, due to its polyanionic nature and lack of specific transporters, phytate is thought to cross the intestinal barrier poorly.^34^ Furthermore, mammalian phytases cytosolic produced such as MINPP1 (Multiple Inositol Polyphosphate Phosphatase 1), can hydrolyse phytate only slowly,^103^ and their expression in intestinal epithelial cells remains uncertain.

While many *in vivo* studies fail to replicate the direct chemical effects of phytate predicted from *in vitro* work, microbial fermentation of phytate to SCFAs has been confirmed in both experimental settings. These findings suggest that the gut microbiota may play a role in mediating the health benefits of dietary phytate. In mice, most phytate appears to be metabolized by gut bacteria,^59^ with only small amounts entering circulation as partially dephosphorylated inositol phosphates or free inositol.^50^ Earlier studies found that both inositol and phytate supplementation reduced hepatic lipogenesis in mice fed a high-sucrose diet, suggesting microbial conversion of phytate to *myo*-inositol as a potential mechanism.^104^

Notably, the gut microbiome can *convert* phytate to lower inositol phosphates such as inositol trisphosphate (InsP₃), which has been shown to promote epithelial repair^105^ and reduce intestinal infection in mice.^106^ For example, *B. thetaiotaomicron* produces a phytase within outer membrane versicles, facilitating InsP_3_ production and subsequent calcium signaling in intestinal enterocytes.^82^ Notably, recent studies have demonstrated that microbial fermentation of phytate enhances the production of SCFAs such as butyrate and propionate,^59^^,^^64^ which are key modulators of glucose metabolism and adiposity.^7^ Supporting this, bacterial supernatants from phytate-fermenting cultures were found to improve barrier function in Caco−2 cell models,^59^ the disruption of which is implicated in systemic inflammatory and infectious consequences associated with obesity and diabetes.^107^ The health benefits of dietary phytate are likely to be partly mediated by SCFA formation through microbial conversion. Moreover, dietary phytate may contribute additional benefits via the production of antimicrobial compounds such as 3-hydroxypropionate and inositol phosphate derivatives. These mechanisms highlight the multifaceted roles of phytate metabolism in shaping host health and underscore the need for further detailed investigations. Taken together, the health benefits of dietary phytate are likely mediated not only by microbial conversion into SCFAs but also through additional metabolites such as 3-hydroxypropionate and inositol phosphate derivatives. These findings highlight the multifaceted nature of phytate metabolism and its potential impact on host physiology. Future studies should therefore aim to disentangle these pathways and establish their specific contributions to health outcomes.

On the other hand, phytate has long been characterized as an antinutrient due to its strong mineral-binding capacity.^108^ For example, zinc absorption was shown to be dose-dependently inhibited by phytate when it was supplemented with meals.^109^ However, such effects may not translate directly to phytate naturally present in foods like cereals and legumes. Additionally, phytate has demonstrated anticancer effects, largely by regulating cell proliferation.^110^ It is evident that further research is needed to clarify the nutritional significance and health impacts of dietary phytate and its derivatives. Recent findings underscore the importance of microbial metabolism in shaping these effects, including the potential liberation of minerals, phosphates, proteins, and lipids otherwise bound by phytate.^59^ Thus, by fermenting phytate, the gut microbiome may enhance nutritional bioavailability and mediate important host–microbe interactions relevant to human health. Future studies are warranted to dissect these mechanisms in greater depth, particularly the role of microbial phytate metabolism in health and disease.

## Conclusions and future perspectives

Inositols and inositol phosphates are abundant dietary components that are associated with benefits to host immunity and metabolism. Emerging evidence suggests that these effects may not arise from direct absorption and action on target tissues, particularly in the case of phytate. Due to its highly polyanionic structure and lack of specific transporters, phytate is poorly absorbed in the gastrointestinal tract. This observation is supported by evidence demonstrating the gut microbiota’s efficient capacity to degrade dietary phytate, likely reflecting an evolutionary adaptation that allows gut microbes to utilize otherwise inaccessible nutrients for their own growth and survival. The microbial conversion of phytate to SCFAs, 3-hydroxypropionate and inositol phosphates may play a pivotal role in mediating the host benefits attributed to phytate and inositol.

Further investigation into phytate-degrading microbial communities, especially within specific subpopulations such as vegetarians or individuals experiencing malnutrition, could guide the development of microbiome-targeted strategies to address mineral deficiencies and malnutrition. It is noteworthy that several phytate-degrading microorganisms can grow under low pH conditions, resembling those of the small intestine, where the majority of minerals and microelements are absorbed. Although concerns remain regarding the inhibitory effects of phytate on the absorption of minerals such as zinc and iron, the gut microbiome may represent a promising therapeutic target to mitigate these antinutritional effects by facilitating mineral release and absorption.
